# A dietary carotenoid reduces immunopathology and enhances longevity through an immune depressive effect in an insect model

**DOI:** 10.1038/s41598-017-12769-7

**Published:** 2017-09-29

**Authors:** Julien Dhinaut, Aude Balourdet, Maria Teixeira, Manon Chogne, Yannick Moret

**Affiliations:** UMR CNRS 6282 BioGéoSciences, Équipe Écologie Évolutive, Université Bourgogne-Franche Comté, Dijon, France

## Abstract

Immunopathology corresponds to self-damage of the inflammatory response, resulting from oxidizing molecules produced when the immune system is activated. Immunopathology often contributes to age-related diseases and is believed to accelerate ageing. Prevention of immunopathology relies on endogenous antioxidant enzymes and the consumption of dietary antioxidants, including carotenoids such as astaxanthin. Astaxanthin currently raises considerable interest as a powerful antioxidant and for its potential in alleviating age-related diseases. Current *in vitro* and short-term *in vivo* studies provide promising results about immune-stimulating and antioxidant properties of astaxanthin. However, to what extent dietary supplementation with astaxanthin can prevent long-term adverse effects of immunopathology on longevity is unknown so far. Here, using the mealworm beetle, *Tenebrio molitor*, as biological model we tested the effect of lifetime dietary supplementation with astaxanthin on longevity when exposed to early life inflammation. While supplementation with astaxanthin was found to lessen immunopathology cost on larval survival and insect longevity, it was also found to reduce immunity, growth rate and the survival of non immune-challenged larvae. This study therefore reveals that astaxanthin prevents immunopathology through an immune depressive effect and can have adverse consequences on growth.

## Introduction

Immunopathology is a remarkably common cause of disease resulting from inflammatory responses of the innate immune system elicited by trauma or infection^1–3^. Inflammation is a phenomenon known from both vertebrates^4^ and invertebrates^5,6^, corresponding to a fast but non-specific response characterised by the delivery of fluids, cytotoxic chemicals and cells to damaged and infected tissues, in order to combat infectious agents and initiate tissue repair. Cytotoxic chemicals released at the focal site of injury or infection comprise highly reactive oxygen species (ROS) and nitrogen species (RNS) destructive to both pathogens and hosts, leading to immunopathology^7^. When damaged tissues are not fully repaired and that homeostasis is not restored, inflammation can further develop into a chronic condition, with inevitable long-term debilitating consequences, such as increased rates of morbidity and mortality at older age^8–10^.

As defence mechanisms, organisms produce a number of endogenous antioxidants capable of scavenging these harmful free radicals and prevent an imbalance between pro- and anti-inflammatory status. However, under conditions of high oxidative stress, the ability of these antioxidants to eliminate free radicals are often exceeded and, therefore, dietary sources of antioxidants are required^11^. These mostly include vitamin E (tocopherol), vitamin C (ascorbate), polyphenolic antioxidants, and carotenoid pigments, which animals obtain from food^12,13^. Carotenoids were reported in many physiological functions, with beneficial effects on survival, growth and immunity^14,15^. They have the ability to scavenge free radicals produced by immune activity^16,17^, and the potential to interact with endogenous antioxidant enzymes^18–20^. By contrast, carotenoids were also suggested to have detrimental effects^21^. Such negative effects were reported on skeletal muscles and reproduction of birds when provided at high doses, mainly under relatively non-stressful conditions, suggesting context-dependent effects of carotenoids^22,23^. Beneficial effects of carotenoids were often attributed to the conversion of these pigments such as β carotene into vitamin A. However, similar effects were found using nonprovitamin A carotenoids such as astaxanthin^11,15,24^. Astaxanthin is a xanthophyll carotenoid mainly produced by fungi and algae, acquired and stored in large amount by aquatic animals, in which the pigment enhances immune activity and limits short term immunopathology effects^20,25,26^. Astaxanthin currently raises considerable interest as a powerful antioxidant and for its potential in alleviating age related diseases^11,15,24,27^. Limiting immunopathology to prevent its negative long-term consequences is currently an important contemporary health issue. For instance, it has been proposed that reduced inflammatory exposure during childhood may have contributed to increased lifespan in human industrialized societies^8^.

While numerous studies support that astaxanthin might be beneficial against immunopathology^11,15,24,27^, yet no study has actually assessed experimentally to what extent dietary supplementation with such a pigment prevents adverse effects of immunopathology on longevity. Insect models offer a great opportunity for such an experiment because they can be easily assessed in large numbers for their whole lifespan in highly controlled laboratory conditions for their diet and immune status. They were therefore proposed as useful model organism to screen for dietary effects on health with relevance for stress resistance and lifespan^28^. The immune system of insects is innate, comprising constitutive defences relying on hemocyte immune cells and several rapidly activated enzyme cascades such as the prophenoloxidase cascade that is at the core of the inflammatory response^5,6,29,30^. Upon infection, hemocytes produce ROS and RNS, which while participating in parasite killing can damage a large range of molecules in cells, inducing apoptotic or necrotic cell death^31–33^. Phenoloxidase enzymes catalyse the formation of toxic quinone intermediates, which undergo further non-enzymatic reactions to form melanin that heals wounds, immobilises invading microbial pathogens through clotting, and encapsulates pathogens in melanised immune cells^29^. Melanin production is also accompanied by the production of ROS and RNS, helping to kill invading organisms^29,31,34,35^. However, such an immune response was also shown to cause damage to self-tissues and organs in the mealworm beetle, *Tenebrio molitor* ^36^. The immunopathology resulting from such an immune response early in the life of the mealworm beetle was also found to reduce longevity^7,37^.

Using *T. molitor* as biological model, we tested whether lifetime food supplementation with astaxanthin helps insects to reduce immunopathology costs in the short and the long term on survival after being exposed to early life inflammation. Mealworm beetles are originally notorious scavengers and decomposers living in leaf-litter and under rocks. They mostly fed on fungi and yeasts growing on decaying vegetables and other organic matter^38^ among which some are producing astaxanthin (e.g., *Phaffia sp*. or *Xanthophyllomices sp*.)^39^. Fresh water microalgae colonizing temporary puddles (e.g., *Haemoatococcus sp*.)^40^ might also be a source of the pigment that the beetles may consume by drinking water or when grazing remains of the algae when the puddle has dried. Nevertheless, the frequency and amount of astaxanthin mealworm beetles may get from their food or drinking water are currently unknown. In this study, we first assessed the phenotypic impact of a controlled immune challenge performed at the larval stage by injection of an inactivated bacterium on larval survival, larval growth and adult longevity of supplemented and non-supplemented insects with astaxanthin. Assuming a strong antioxidant effect of astaxanthin, thus reducing the costs of immunopathology, we predicted that lifetime food supplementation should have positive effects on larval survival after the immune challenge, insect growth and adult longevity. By contrast, assuming context dependent effects of carotenoids, detrimental effects of the food supplementation with astaxanthin might also be observed among non-immune-challenged insects. In addition, since carotenoids were often reported to have a broad immune stimulating effect, we further examined the influence of food supplementation with astaxanthin on important cellular and humoral immune effectors in larvae, after a controlled immune challenge. We also further tested whether food supplementation with astaxanthin improves the resistance of larvae to an infection with living bacterial pathogens in survival experiments. Assuming a general immune stimulating effect of astaxanthin, food supplementation with this pigment was expected to increase levels of immune defence and resistance to infection.

## Results

### Larval survival, growth and longevity after an immune challenge

To know whether astaxanthin has indeed the potential to prevent immunopathology costs and improve longevity, we first tested experimentally whether dietary supplementation with this pigment influences larval survival, larval development and insect longevity after being subjected to an immune challenge. To this purpose, 9 weeks old larvae that were supplemented or not with astaxanthin for 3 weeks, and for which the food treatment was carried on for the entire life of the insects, were either or not immune challenged with a suspension of inactivated *Bacillus thuringiensis*, mimicking a bacterial infection and stimulating the immune response.

**Figure 1:**
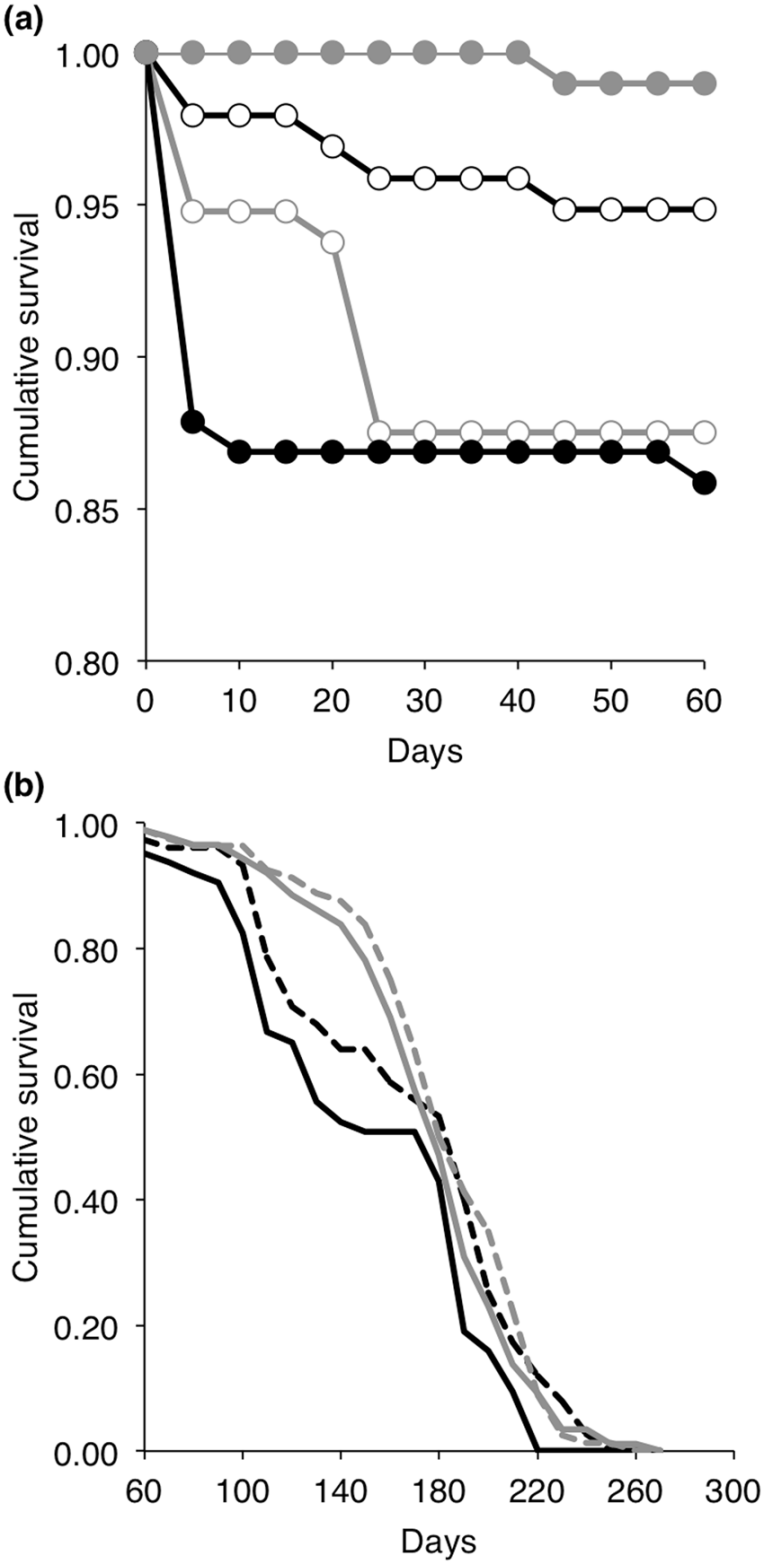
Larval survival (**a**) and whole insect longevity (**b**) of supplemented (grey lines) and non-supplemented (black lines) insects with astaxanthin after being exposed to an immune challenge by injection of a suspension of inactivated *Bacillus thuringiensis* (5 µL, 10^8^ cells.mL^−1^). Larval survival is shown for larvae that were exposed (filled circles) or not (opened circles) to an immune challenge (**a**), whereas whole insect longevity is shown for males (dashed lines) and females (continuous lines) for which the immune challenge had no significant influence (**b**).

Survival of the larvae was found dependent on the interaction between the dietary supplementation and the immune challenge (Table 1). While the food treatment had no main statistical effect on survival on its own, the immune challenge marginally reduced larval survival (Table 1). To explain such a statistical interaction between the dietary treatment and the immune challenge, we tested the effect of the immune challenge on survival for larvae supplemented and non-supplemented separately. Among non-supplemented larvae, the immune challenge was associated with a 4 folds survival reduction (W = 8.90, p = 0.003, Odd ratio = 4.40, n = 195, Fig. 1a), whereas the immune challenge slightly improved the survival of supplemented larvae with astaxanthin (W = 4.83, p = 0.028, Odd ratio = 0.35, n = 195, Fig. 1b).

**Table 1:** Results of time-dependent Cox regression analyses for larval survival and the whole longevity of *Tenebrio molitor* (n = 385) according to food supplementation with asatxanthin (Food) and the immune challenge (Challenge).

*Larval survival*						
Variables in the best model	B	s.e.	Wald	df	p	Odd ratio
Challenge	0.73	0.38	3.63	1	0.057	2.07
Food * Challenge	-2.58	0.69	14.15	1	** < 0.001**	0.76
Challenge * T-Cov	-0.04	0.02	3.19	1	0.074	0.96
**Variables not in the best model**	**Score**	**df**	**p**			
Food	0.94	1	0.760			
Food * T-Cov	0.23	1	0.634			
Challenge * Food * T-Cov	0.36	1	0.549			
***Whole insect survival***						
**Variables in the best model**	**B**	**s.e**.	**Wald**	**df**	**p**	**Odd ratio**
Food	−1.53	0.46	10.87	1	**0.001**	0.22
Sex	−0.23	0.12	4.88	1	**0.027**	0.772
Food * T-Cov	0.008	0.003	8.16	1	**0.004**	1.01
**Variables not in the best model**	**Score**	**df**	**p**			
Challenge	1.15	1	0.284			
Challenge * Food	0.15	1	0.696			
Food * Sex	1.29	1	0.253			
Challenge * T-Cov	0.66	1	0.416			
Challenge * Sex	0.44	1	0.506			
Sex * T-Cov	0.46	1	0.498			
Challenge * Food * T-Cov	0.001	1	0.997			
Food * Sex * T-Cov	1.53	1	0.216			
Challenge * Sex * T-Cov	0.32	1	0.568			

A time-dependent covariate (T-Cov.) was specified and included in interaction with the explanatory variables to account for their time-dependent effect. The “simple” contrast was used for Food (survival of non-supplemented larvae was used as baseline), and challenge (survival of larvae injected with saline solution only was used as baseline). The best model was searched using backward stepwise method utilizing likelihood ratio significance tests for evaluation of each effect. Procedure is available in COXREG procedure of SPSS statistical package. Model fitting was initiating with a model that included all main effect and two ways interactions, with the exception of Box. Values p ≤ 0.05 are given in bold.

Beyond the larval stage, insect longevity was significantly increased by the supplementation with astaxanthin, whereas it was unaffected by the immune challenge (Table 1, Fig. 1B). The positive effect of the dietary pigment on insect longevity slightly declined with time (see Food * T-Cov in Table 1). Male insects were more long-lived than females, independently of the immune challenge or their diet (Table 1, Fig. 1b).

**Figure 2:**
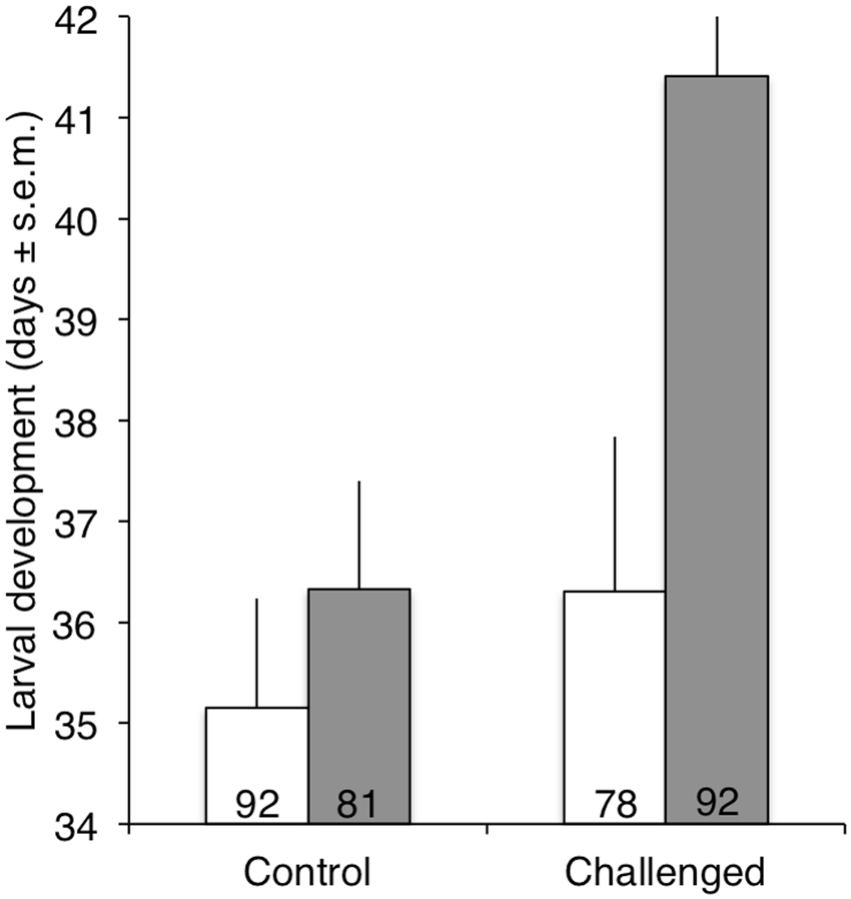
Larval development time in days of supplemented (grey bars) and non-supplemented (white bars) insects with astaxanthin after being exposed to an immune challenge by injection of a suspension of inactivated *Bacillus thuringiensis* (5 µL, 10^8^ cells.mL^−1^). Numbers at the bottom of the bars refer to sample size.

Larval developmental time of insects that reached the adult stage was prolonged by both the supplementation with astaxanthin and the immune challenge in an additive manner (Table 2; Fig. 2). However, mass values of the resulting nymph and then adult were not affected by the dietary treatment or the immune challenge (Table 2).

**Table 2:** Multivariate analysis of variance for development time, nymph mass and adult mass of mealworm beetle larvae (n = 335) as a function of food and immune treatments.

Source of Variation		df	F	p
MANOVA (Pillai’s trace)	Food	3, 330	3.70	**0.012**
	Challenge	3, 330	2.96	**0.033**
ANOVA development time	Global model	3, 332	7.74	**0.001**
	Food	1, 332	6.76	**0.010**
	Challenge	1, 332	7.37	**0.007**
ANOVA nymph mass	Global model	3, 332	0.77	0.463
	Food	1, 332	1.19	0.276
	Challenge	1, 332	0.25	0.618
ANOVA adult mass	Global model	3, 332	0.20	0.816
	Food	1, 332	0.01	0.931
	Challenge	1, 332	0.41	0.525

Food and immune treatments had no interactive effect on the above parameters and were consequently removed from the statistical model. The multivariate test is shown first, followed by the respective univariate tests for development time, nymph mass and adult mass. Values p ≤ 0.05 are given in bold.

### Carotenoids and Immunity after an immune challenge

**Figure 3:**
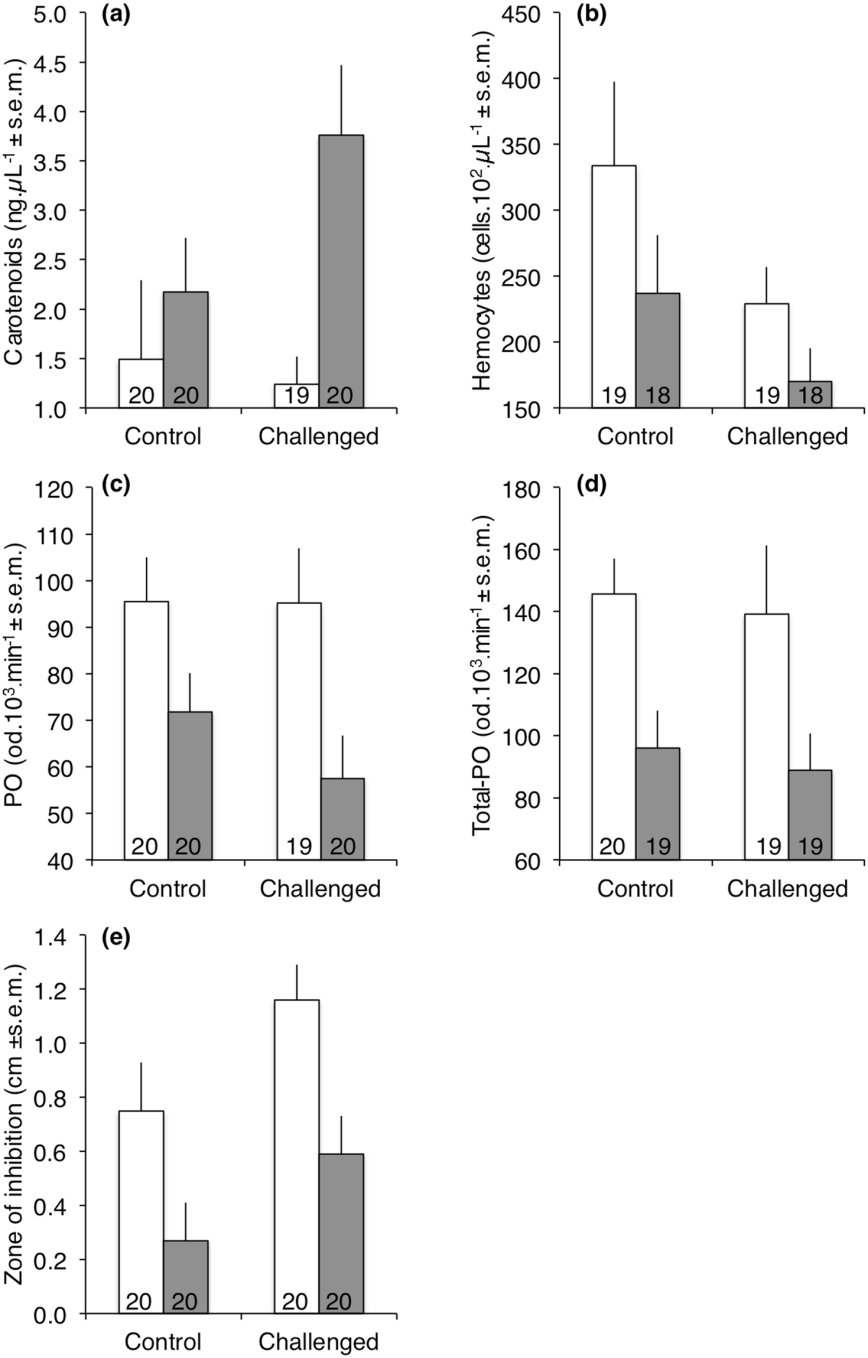
Concentration of carotenoids (**a**), concentration of hemocytes (**b**), PO activity (**c**), Total-PO activity (**d**) and antibacterial activity (**e**) in the hemolymph of supplemented (grey bars) and non-supplemented (white bars) larvae with astaxanthin after being exposed to an immune challenge by injection of a suspension of inactivated *Bacillus thuringiensis* (5 µL, 10^8^ cells.mL^−1^). Numbers at the bottom of the bars refer to sample size.

From the above experiment, we also measured levels of circulating carotenoids in the hemolymph and examined potential changes in immune defences resulting from both the dietary supplementation and the immune treatment of the larvae three days after their immune challenge occurred. Many of these variables were influenced by both the dietary and immune treatments, but not their interaction (Table 3). More specifically, dietary supplementation with astaxanthin significantly increased by 2 folds the circulating concentration of carotenoids in the hemolymph of *T. molitor* larvae after 3 weeks of food treatment (Table 3, Fig. 3a). However, the immune challenge had no influence on the concentration of carotenoids (Table 3). All the immune parameters measured were influenced by the dietary treatment (Table 3). Overall, the supplemented larvae with astaxanthin exhibited lower hemocyte concentration (Table 3, Fig. 3b), lower activity of the phenoloxidase system (Table 3, Fig. 3c, d) and lower antibacterial activity (Table 3, Fig. 3e) than non-supplemented larvae. Only antibacterial activity was found significantly affected by the immune challenge (Table 3). Indeed, unsurprisingly, the bacterial immune challenge resulted in an induced antibacterial response, which is known to last several days^41^. Hemocyte concentration tended to decrease after the immune challenge, but this effect was marginally significant (Table 3).

**Table 3:** Multivariate analysis of variance for carotenoid concentration, hemocyte concentration, PO activity, total-PO activity and antibacterial activity of mealworm beetle larvae (n = 144) as a function of food treatment and immune treatment.

Source of Variation		df	F	p
MANOVA (Pillai’s trace)	Food	5, 65	5.32	**<0.001**
	Challenge	5, 65	3.37	**0.009**
ANOVA Carotenoid	Global model	2, 69	3.54	**0.035**
	Food	1, 69	4.99	**0.029**
	Challenge	1, 69	2.08	0.154
ANOVA Hemocyte	Global model	2, 69	3.94	**0.024**
	Food	1, 69	4.32	**0.041**
	Challenge	1, 69	3.56	0.063
ANOVA PO activity	Global model	2, 69	4.88	**0.010**
	Food	1, 69	8.81	**0.004**
	Challenge	1, 69	0.96	0.331
ANOVA total-PO activity	Global model	2, 69	5.21	**0.008**
	Food	1, 69	10.00	**0.002**
	Challenge	1, 69	0.43	0.516
ANOVA antibacterial activity	Global model	2, 69	8.14	**0.001**
	Food	1, 69	9.31	**0.003**
	Challenge	1, 69	6.96	**0.010**

Food and challenge treatments had no interactive effect on the above parameters and was consequently removed from the statistical model. The multivariate test is shown first, followed by the respective univariate tests for carotenoid concentration, hemocyte concentration, PO activity, total-PO activity and antibacterial activity. Values p ≤ 0.05 are given in bold.

### Survival to bacterial infection

**Figure 4:**
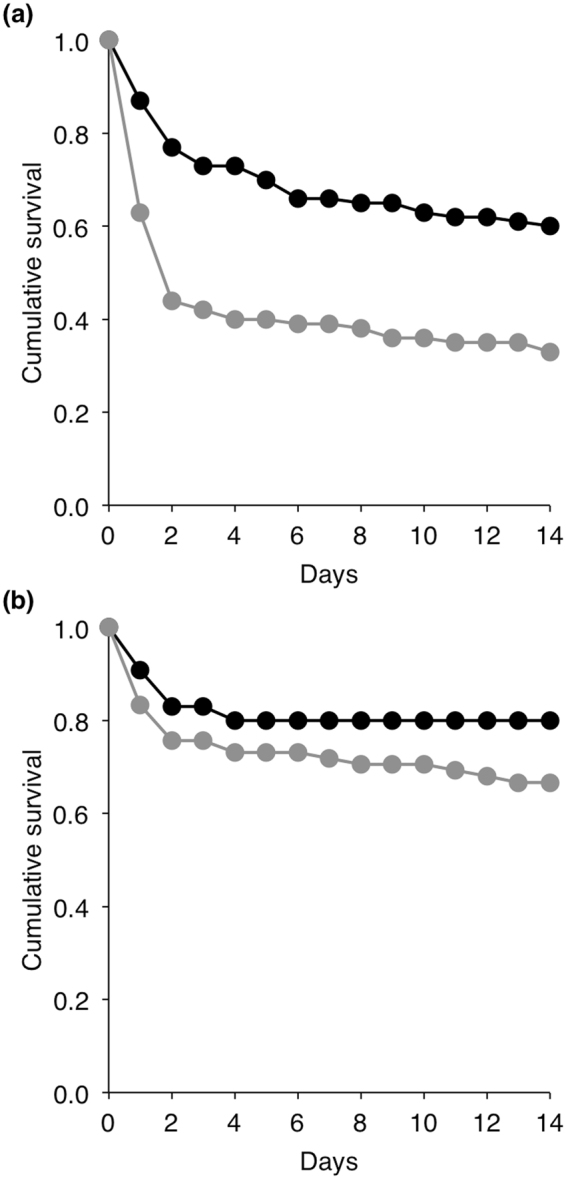
Survival of supplemented (grey lines) and non-supplemented (black lines) larvae with astaxanthin after being exposed to an infection with either (**a**) *Bacillus cereus* or (**b**) *Bacillus thuringiensis*.

Because of the apparent immune depressive effect of astaxanthin observed in the above experiment, we investigated whether the supplementation with astaxanthin affect the susceptibility of insect larvae to a bacterial infection using two known entomopathogenic bacterial pathogens of the mealworm beetle, *Bacillus cereus* and *B. thuringiensis* ^42^. Dietary supplemented larvae with astaxanthin were more sensitive to the infection with *B. cereus* than non-supplemented larvae (Cox regression: W = 16.19, p < 0.001 Odd ratio = 2. 25, N = 200, Fig. 4a). Similarly, dietary supplemented larvae with astaxanthin were slightly more sensitive to the infection with *B. thuringiensis* than non-supplemented ones, but this survival difference was only marginal (W = 2.91, p = 0.088, Odd ratio = 1. 79, N = 143, Fig. 4b).

## Discussion

This study tested whether lifetime food supplementation with an important antioxidant can prevent short and long-term immunopathological consequences of early life inflammation in the mealworm beetle, *T. molitor*. Because astaxanthin is predicted to have a strong antioxidant activity^15,20,24,26^, lifetime food supplementation with that pigment was expected to improve larval survival and adult longevity of the insects exposed to an immune challenge at the larval stage. By contrast, since carotenoids, including xanthophylls, are also believed to induce context dependent detrimental effects^21–23^, food supplementation with astanxanthin might also be associated to negative effects among non-immune-challenged insects.

Dietary supplementation with astaxanthin significantly increased by 2 folds the circulating concentration of carotenoids in the hemolymph of *T. molitor* after 3 weeks of food treatment. However, the amount of circulating carotenoids in the hemolymph was still relatively low compared to the amount of astaxanthin provided to supplemented insects. Furthermore, the circulating concentration of carotenoids in the hemolymph of supplemented *T. molitor* larvae with astaxanthin was about 400 times lower than the one found in the hemolymph of the freshwater crustacean, *G. pulex*, similarly supplemented in laboratory conditions^26^. In the present study, considering the bright red colour of the insect faeces, large amounts of the pigment were apparently not absorbed.

Food supplementation with astaxanthin induced contrasted results for survival under immune stimulation with inactivated bacteria (i.e. inducing an immune response without any pathogenic effect due to pathogen virulence). While supplementation with astaxanthin in larvae was associated with a survival benefit under immune stimulation, it was also associated with a survival reduction in absence of immune challenge. The survival benefit under immune challenge is consistent with the results of a previous study that tested the survival cost of an immune response produced by supplemented and non-supplemented gammarid crustaceans with a mix of astaxanthin and lutein^26^. The strong antioxidant property of astaxanthin enabling the capture of cytotoxic free radicals produced by immune activity^16,17^ and its ability to stimulate enzymes of the endogenous antioxidant defence system^20^ might have contributed to limit negative effects of the immune response by autoreactivity. However, an important result of this study is that supplementation with astaxanthin is also associated to a reduced immune activity, even upon an immune challenge with inactivated bacteria. Indeed, insects fed with astaxanthin exhibited reduced hemocyte concentration, lower levels of phenoloxidase activity and low synthesis of antibacterial activity upon a bacterial immune challenge. This further caused an increased susceptibility to infection with bacterial entomopathogens. These results clearly contrast with the general belief that carotenoids have immune stimulating properties. Indeed, supplementation with astaxanthin was reported to stimulate some markers of immunity in vertebrates^15,24^ and invertebrates, mainly crustaceans^14,20,26,43–45^. For instance, in the amphipod crustacean *Gammarus pulex*, experimental dietary supplementation with astaxanthin results in broad stimulation of gammarid innate immune defences, giving rise to increased resistance to microbial infection^26^. Similarly, astaxanthin dietary supplementation increases phenoloxidase activity and total hemocyte count in the giant freshwater prawn *Macrobrachium rosenbergii* ^44^. In the same species, injection of astaxanthin increases total hemocyte count and survival in presence of the pathogenic bacterium *Lactococcus garvieae* ^43^. Since crustaceans have evolved particular carotenoprotein complexes allowing the storage of large amount of astaxanthin in their tissues^45^, this pigment might be of particular importance in their physiology, including immunity.

By contrast, *T. molitor* is not known to possess specialized features to store carotenoids and the supplementation of the food with astaxanthin is found here associated to a general down regulation of its innate immune system. Such a general immune depressive effect of astaxanthin may rely on the interaction of the pigment with the availability or production of nitric oxide (NO), which has been evidenced to be a major regulator of the insect immune response. Indeed, NO has been found to stimulate both cellular and humoral immunity of insects^46–49^, and astaxanthin may affect the availability of NO in two ways. First, astaxanthin was reported to inhibit the activity of the nitric oxide synthase, the enzyme responsible of NO production from L-arginin^24^. Second, because of its strong antioxidant power, astaxanthin might also have interfered with NO cellular signalling, by scavenging a certain fraction of circulating NO, thus down regulating base levels of immune activity. Another but not exclusive explanation is that astaxanthin may also have regulatory effects on the host’s metabolism^50,51^. Recently, astaxanthin was found to interact with nuclear receptors of the peroxisome proliferator-activated receptor superfamily, which regulates lipid and glucose metabolism in vertebrates^52^. Such an alteration of the host metabolism could reduce the allocation of energetic resource to the immune system. If these receptors are conserved among taxa, similar regulatory effects may occur as well in invertebrates. Whatever the mechanisms by which astaxanthin may down regulate the immune system of *T. molitor*, its immune depressive effect might have been a major cause of the reduced cost of the immune response to inactivated bacteria on larval survival. Furthermore, resources saved from reduced immune activity may also have contributed to the prolonged longevity of supplemented beetles, independently of the immune challenge at the larval stage.

The slight, but significant, survival cost in absence of immune challenge also confirms previous observations showing detrimental effects of carotenoid supplementation in non-stressed birds^22,23^, although cautions should be taken about comparisons drawn from different taxa. In addition, the supplementation with astaxanthin was also associated to a prolonged larval development of the beetles, although not affecting adult body size. Available data show that astaxanthin inhibits cell proliferation and induces enhanced apoptosis activity^53,54^. Apoptosis corresponds to an essential programed cell death occurring during the normal development of multicellular organisms^55^. Its activity is usually balanced with cell proliferation, ensuring normal growth and survival. By concomitantly promoting apoptosis and inhibiting cell proliferation, astaxanthin might have constrained the normal growth of the developing mealworm larvae, leading to the observed prolonged larval development in supplemented insects. This dual effect of astaxanthin may also have contributed to the slight increase of mortality among supplemented larvae that were not immune-challenged. The modulation of apoptosis by carotenoids appears to be variable according to several factors such as carotenoids concentration and antioxidant status^53^. The immune challenge of the larvae is believed to promote a pro-oxidant status by the release of oxidative free radicals^31–33^. The mobilization of astaxanthin for the detoxification of free radicals produced during the immune response may have relieve the negative impact of the pigment on the survival of the larvae, and could explain why immune-challenged larvae exhibited the highest survival among supplemented larvae. Further detailed analysis would be needed to test this hypothesis.

To summarize, we found that life-time supplementation of *T. molitor* with astaxanthin, an important dietary antioxidant, can prevent early and late immunopathology costs, which result in a better tolerance to immune cost at the larval stage and a prolonged longevity. However, while these beneficial effects might, to some extent, directly result from the strong antioxidant property of the dietary pigment, its strong down regulating effect on the insect immune system is likely to be a major cause. This immune depressive effect of astaxanthin has also the disadvantage of decreasing the insect resistance to bacterial infection. Other detrimental effects of the supplementation with astaxanthin could be revealed on survival of non-stressed larvae and on larval development of the insects. This study suggests that dietary carotenoids could be challenging for biological systems, at least for those that did not evolve specialized features to store them, and that beneficial and detrimental effects resulting from the supplementation with these pigments might be host specific and context dependent. While Dual effects of carotenoids will have to be considered for their use in the development of products promoting health.

## Material and Methods

### Insect cultures

Experimental insects were produced in routine by allowing groups of 10 days old virgin adult beetles (10 males and 10 females) to reproduce 3 days in plastic boxes (L × 1 × H, 20 × l2 × 9.5 cm) supplied with 60 g of bran flour, a micro centrifuge tube of water in standard laboratory conditions (25 °C, 70% RH; 24 h dark). Parental insects where then removed and eggs produced in each box were allowed to develop. Six weeks after egg laying, the offspring larvae obtained in each box were counted and their number adjusted to 30 larvae per box, and provided with fresh bran flour. Half of the boxes were allocated to dietary supplementation with astaxanthin whereas the other half of the boxes was allocated to the control food treatment. Each box of supplemented insects was provided twice a week with micro 0.5 mL centrifuge tubes (3 per box) containing 500 µL of a solution of astaxanthin (Carophyll Pink® 10%, 30 mg per mL of distilled water in 1% mass/vol. of agar), corresponding to 1.5 mg of astaxanthin twice a week (or about 0.1 mg of astaxanthin per larvae and per week), whereas each control boxes were similarly provided with micro centrifuge tubes of distilled water in 1% agar only, for the whole duration of the experiments. Insects were used for the experiments three weeks after the start of the dietary supplementation.

The first experiment testing for larval survival, growth and insect longevity after an immune challenge used 8 boxes of each dietary treatment (total of 16 boxes), each containing 30 nine-weeks old larvae. Half of the boxes within each dietary treatment group were allocated to an immune challenge mimicking a bacterial infection, whereas the other half of the boxes was allocated to a control immune treatment. Challenged larvae were injected with a 5-µL suspension of inactivated *B. thuringiensis* (10^8^ bacteria.mL^−1^) in phosphate buffer saline (PBS 10 mM, pH 7.4) corresponding to a non deadly dosage previously used to characterize the immune response of the mealworm beetle^56,57^. Control larvae were treated in the same way, but without bacteria, as a procedural control. Three days later, five larvae per box were randomly taken to collect a 5 µL-sample of haemolymph to measure astaxanthin concentration, haemocyte concentration, antibacterial activity and the maintenance and use of the prophenoloxidase system. After sampling, these larvae were not returned into the experimental cultures. Starting from the immune treatment of the larvae, each box was checked twice a week to record larval survival, larval developmental time (duration in days from hatching to adult), nymph body mass, adult body mass and total longevity of the remaining insects. As soon as larvae reached the pupae stage, they were weighed and allowed achieving their live span isolated in grid boxes (boxes with 10 compartments; each compartment: L × 1 × H, 4.8 × 3.2 × 2.2 cm) supplied with bran flour and their respective dietary treatment.

The experiments testing the susceptibility of supplemented and non-supplemented larvae to the infection by *B. cereus* consisted of the inoculation of 100 supplemented larvae with astaxanthin and 100 non-supplemented ones with a fine sterilized needle dipped into a pellet of live bacteria. The infection experiment using *B. thuringiensis* used exactly the same procedure, in which 78 supplemented larvae with astaxanthin and 65 non-supplemented larvae were inoculated. Larvae were kept individually in grid boxes supplied with bran flour and their respective dietary treatment. Survival to infection was recorded once a day for 14 days.

### Bacterial culture for immune challenge and infections

The bacteria used in this study are known to be pathogens of *T. molitor* ^42^. *B. thuringiensis* and *B. cereus* were obtained from the Pasteur institute: *B. thuringiensis* (CIP53.1); *B. cereus* (CIP69.12). Bacteria were grown overnight at 28 °C in liquid Broth medium (10 g bacto-tryptone, 5 g yeast extract, 10 g NaCl in 1000 mL of distilled water, pH 7). Bacteria used to performed immune challenges were then inactivated in 0.5% formaldehyde prepared in PBS for 30 minutes, rinsed three times in PBS, and their concentration adjusted to 10^8^ bacteria per mL using a Neubauer improved cell counting chamber^56,57^. The success of the inactivation was tested by plating a sample of the bacterial solution on sterile Broth medium with 1% of bacterial agar and incubated at 28 °C for 24 hours. Aliquots were kept at -20 °C until use. After being chilled on ice for 10 min for immobilization, insects were immune challenged by injection of 5 µL of the bacterial suspension through the pleural membrane between the second and third abdominal segment using sterile glass capillaries that had been pulled out to a fine point with an electrode puller (Narashige PC-10). For bacteria used for insect infection, overnight bacterial cultures (20 mL) were centrifuged at 3500 g at 4 °C for 30 min. The supernatant was discarded and the bacteria pellet was used for infection. After being chilled on ice for 10 min, insects were infected by dipping a sterilized 0.03 mm diameter needle (Fine Science Tools® n° 26000-25) into the bacteria pellet and pricking the animal through the pleural membrane between the second and third abdominal segment.

### Hemolymph collection, astaxanthin dosage and immune parameters

Hemolymph was collected as described by Moret^58^. After being chilled on ice for 10 min for immobilization, each larvae provided 5 µL of hemolymph collected into a sterile pre-chilled 5 µL-graduated glass capillary (Ringcaps®, Hirshmann® Laborgerate, Germany) after wounding the insect between the second and the third abdominal segments with a sterile needle. The sample of hemolymph was immediately diluted in 30 µL of ice-cold PBS. A first 10-µL subsample was immediately used for the measurement of the concentration of hemocytes, using a Neubauer improved cell counting chamber under a phase-contrast microscope (magnification × 400). A second 10-µL subsample was frozen in liquid nitrogen and stored at −80 °C for later estimation of the concentration of astaxanthin. Another 5-µL subsample was transferred into an N-phenylthiourea (Sigma-Aldrich, St Louis, MO, USA, P7629)-coated microcentrifuge tube, frozen in liquid nitrogen and stored at −80 °C until later examination for antibacterial activity. The remaining hemolymph solution was diluted with 10 µL of PBS, frozen in liquid nitrogen and stored à -80 °C for later measurement of the phenoloxidase activity

Carotenoids were extracted and quantified following the method of Cornet and colleagues^25^. Briefly, pigments were extracted by adding the same volume of ethanol and washing pellets twice with 200 µL of methyl-*tert*-butyl ether (MTBE). Via a colorimetric assay, the concentration of pigments was determined at 470 nm in a microplate reader against a reference curve ranging from 0 to 50 ng/µL of a standard solution of astaxanthin in ethanol (standards obtained from Extrasynthèse, Genay, France). Values were corrected to obtain concentrations for 1 μL of pure hemolymph.

Antimicrobial activity in the hemolymph was measured using a standard zone of inhibition assay^58^. Samples were thawed on ice, and 2 µL of the sample solution were used to measure antimicrobial activity on zone of inhibition plates seeded with *Arthrobacter globiformis* from the Pasteur institute (CIP105365). An overnight culture of the bacterium was added to broth medium containing 1% agar to achieve a final concentration of 10^5^ cells per mL. Six millilitres of this seeded medium was then poured into a Petri dish and allowed to solidify. Sample wells were made using a Pasteur pipette fitted with a ball pump. Two microlitres of sample solution were added to each well, and a positive control (Tetracycline: Sigma-Aldrich, St Louis, MO, USA, T3383; 2.5 mg.mL^−1^ in absolute ethanol) was included on each plate^59^. Plates were then incubated overnight at 28 °C. Then, the diameter of inhibition zones was measured for each sample.

For each individual hemolymph sample, both the activity of naturally activated phenoloxidase (PO) enzymes only (PO activity), and the activity of the proenzymes (proPO) in addition to that of the PO (total-PO activity), were measured using a spectrophotometer^50^. The PO activity was quantified without further activation, while the total-PO activity required the activation of the proPO into PO with chymotrypsin. For this purpose, frozen hemolymph samples were thawed on ice and centrifuged (3500 g, 5 min, 4 °C). Five microlitres of supernatant were added to a microplate well containing 20 µL of PBS, and either 140 µL of distilled water to measure PO activity only, or 140 µL of chymotrypsin solution (Sigma-Aldrich, St Louis, MO, USA, C-7762, 0,07 mg.mL^−1^ of distilled water) to measure total-PO activity. Then 20 µL of L-Dopa solution (Sigma-Aldrich, St Louis, MO, USA, D-9628, 4 mg mL^−1^ of distilled water) was added to each well. The reaction was allowed to proceed at 30 °C in a microplate reader (Versamax; Molecular Devices, Sunnyval, CA, USA) for 40 min. Readings were taken every 15 s at 490 nm and analysed using the software SOFT-Max Pro 4.0 (Molecular Devices, Sunnyval, CA, USA). Enzyme activity was measured as the slope (Vmax value: change in absorbance unit per min) of the reaction curve during the linear phase of the reaction and reported to the activity of 1 µL of pure hemolymph.

### Statistics

Survival of larvae and the whole insect longevity with respect to dietary supplementation with astaxanthin and immune challenge were analysed using time-dependent Cox regression analyses because the proportional hazards assumption was not met (risk of mortality was not constant over time). Boxes in which the larvae were maintained did not explain survival within each treatment combination (non-supplemented and non-challenged larvae: W = 0.76, df = 3, p = 0.858; non-supplemented and challenged larvae: W = 3.01, df = 3, p = 0.390; supplemented and non-challenged larvae: W = 2.21, df = 3, p = 0.530; supplemented and challenged larvae: W = 2.41, df = 3, p = 0.492) and could therefore be ignored in further analysis. Our statistical models used a stepwise procedure and the reference survival functions were generated from the control data derived from the dietary (e.g. non-supplemented) and the immune treatment (e.g. control). Dietary and immune treatments were coded as categorical variables, and insect larvae that reached the adult stage during the survey were censored. Analysis of the whole insect longevity also used sex as categorical explanatory variable. A time-dependent covariate was specified and included in interaction with all explanatory variables to test for their time-dependent effect.

Larval development time, nymph and adult body mass were analysed using a MANOVA with dietary supplementation, immune treatment and sex as factors.

Carotenoid concentration, hemocyte concentration, PO activity, total-PO activity and antibacterial activity were analysed using a multivariate analysis of variance (MANOVA) with dietary and immune treatments as factors. Data on carotenoid concentration and antibacterial activity were natural log transformed whereas those on hemocyte concentration square root transformed to satisfy the requirements of parametric statistical tests.

Survival to bacterial infections with respect to dietary supplementation with astaxanthin was analysed using proportional hazards Cox regressions that used a stepwise procedure and the reference survival functions were generated from the control data derived from the dietary (e.g. non-supplemented).

All statistical analyses used IBM® SPSS® Statistics 19 for Macintosh.

### Data availability

The datasets generated during and/or analysed during the current study are available from the corresponding author on reasonable request.
